# Supplementary material for: Immune phenotypes that predict COVID-19 severity
Source: Res Sq. 2022 Mar 10:rs.3.rs-1378671. Preprint. [Version 1] doi: 10.21203/rs.3.rs-1378671/v1 (PMC8923110; doi:10.21203/rs.3.rs-1378671/v1)
Supplement: Supplement 1 [file 0a36f7cff6ed159a24c59220.pdf]

Supplementary Table 1: Staining Panels

F/S column refers too functional (F) or subset-defining (S) marker. marker was used for both in case of F/S.

TNK backbone			
	**Marker**	**Fluorochrome**	**F/S**
B515	TCR Vd1	FITC	S
B610	CD127	BB630	S
B660	PD-1	BB660	F
B710	CD16	BB700	S
B780	CXCR5	BB790	S
G575	TCR Vg9	PE	S
G610	TCR Vd2	PE-CF594	S
G660	CD161	PE-Cy5	S
G710	HLA-DR	PE-Cy5.5	F/S
G780			
R670	d:PBS57 tetra	APC	S
R730	CD45RA	Ax700	S
R780			
U395			
U450	Violet L/D	UV Blue	
U500	CCR7	BUV496	S
U570	CD56	BUV563	S
U670	CD39	BUV661	F
U740	CD95	BUV737	S
U785	CD4	BUV805	S
V450			
V510	CD3	BV510	S
V570	CD8a	BV570	S
V605	CD38	BV605	F/S
V655			
V710	TCR Va7.2	BV711	S
V750			
V785	CD27	BV786	S

BDC backbone			
	**Marker**	**Fluorochrome**	**F/S**
B515	CADM1	FITC	S
B610	CD141	BB630	S
B660	CD123	BB660	S
B710	FcEr1a	BB700	S
B780	IgD	BB790	S
G575	IFNAR2	PE	F
G610	CD88	PE-Dazzle594	S
G660	CD3	PE-Cy5	S
G710	CD5	PE-Cy5.5	S
G780			
R670	CD11c	APC	S
R730	CD27	APC-R700	S
R780			
U395			
U450	Violet L/D	UV Blue	
U500	CD40	BUV496	F
U570	CD56	BUV563	S
U670	CD21	BUV661	S
U740	CD163	BUV737	S
U785	CD20	BUV805	S
V450			
V510	CD14	BV510	S
V570	CD16	BV570	S
V605	CD38	BV605	S
V655			
V710	CD86	BV711	F
V750			
V785	HLA-DR	BV786	S

Chemokine receptor 1			
	**Marker**	**Fluorochrome**	**F/S**
V450	CCR2	BV421	F
U395	CCR3	BUV395	F
V655	CCR5	BV650	F
V750	CX3CR1	BV750	F
G780	CCR1	PE-Cy7	F
R780	XCR1	APC-Fire750	F

Chemokine receptor 2			
	**Marker**	**Fluorochrome**	**F/S**
V450	CCR9	BV421	F
U395	CCR8	BUV395	F
V655	CCR4	BV650	F
V750	CXCR6	BV750	F
G780	CXCR3	PE-Cy7	F
R780			

Chemokine receptor 2: BDC			
	**Marker**	**Fluorochrome**	**F/S**
R780	CD19	APC-H7	F

Chemokine receptor 2: TNK			
	**Marker**	**Fluorochrome**	**F/S**
R780	TIGIT	APC-Fire750	F

Supplementary Table 2: Staining reagents

Fluorescence channel	Marker	Fluorochrome	Clone	Cat#	Lot#	Manufacturer	Amount for 50ul	Panel
U450	Viability Violet	UV Blue	-	L34962		Thermo Fisher Scientific	0.0641	All samples
B515	TCR Vd1	FITC	TS8.2	TCR2730	TA259706	Thermo Fisher Scientific	2.5	TNK backbone
B610	CD127	BB630	HIL-7R-M21	624294	1028625	BD Biosciences	1.25	TNK backbone
B660	PD-1	BB660	EH12.1	624295	1028640	BD Biosciences	0.31	TNK backbone
B710	CD16	BB700	3G8	746199	8234672	BD Biosciences (OptiBuild)	0.04	TNK backbone
B780	CXCR5	BB790	RF8B2	624296	8241555	BD Biosciences	0.04	TNK backbone
G575	TCR Vg9	PE	B3	555733	0188066	BD Biosciences	1.25	TNK backbone
G610	TCR Vd2	PE-CF594	B6	624352	8241641	BD Biosciences	0.01	TNK backbone
G660	CD161	PE-Cy5	DX12	551138	8180780	BD Biosciences	2.5	TNK backbone
G710	HLA-DR	PE-Cy5.5	TU36	MHLDR18	1990699A	Thermo Fisher Scientific	0.31	TNK backbone
R670	1d:PBS57 tetrar	APC	-	41386	27271P	NIH tetramer core	0.15	TNK backbone
R730	CD45RA	Ax700	HI100	560673	8164502	BD Biosciences	0.63	TNK backbone
U500	CCR7	BUV496	2-L1-A	749827	everal pre-mixe	BD Biosciences	5	TNK backbone
U570	CD56	BUV563	NCAM16.2	565704	8065832	BD Biosciences	0.31	TNK backbone
U670	CD39	BUV661	TU66	749967	everal pre-mixe	BD Biosciences	0.63	TNK backbone
U740	CD95	BUV737	DX27	624286	8242545	BD Biosciences	1.25	TNK backbone
U785	CD4	BUV805	SK3	564910	7270541	BD Biosciences	0.63	TNK backbone
V510	CD3	BV510	UCHT1	563109	8174765	BD Biosciences	0.15	TNK backbone
V570	CD8a	BV570	RPA-T8	301038	B262531	Biolegend	0.267	TNK backbone
V605	CD38	BV605	HIT2	740401	everal pre-mixe	BD Biosciences	1.25	TNK backbone
V710	TCR Va7.2	BV711	3C10	351732	B230086	Biolegend	1.25	TNK backbone
V785	CD27	BV786	L128	624292	8242923	BD Biosciences	0.31	TNK backbone
B515	CADM1	FITC	30	CM004-4	004	MBL International Corporation	0.15	BDC backbone
B610	CD141	BB630	1A4	624294	0345830	BD Biosciences	0.01	BDC backbone
B660	CD123	BB660	7G3	624295	0345827	BD Biosciences	0.04	BDC backbone
B710	FcEr1a	BB700	AER-37	747780	everal pre-mixe	BD Biosciences	0.63	BDC backbone
B780	IgD	BB790	IA6-2	624296	8241553	BD Biosciences	0.31	BDC backbone
G575	IFNAR2	PE	REA124	130-099-555	1320121071	Miltenyi	1.25	BDC backbone
G610	CD88	PE-Dazzle594	S5/1	344318	B318054	Biolegend	0.31	BDC backbone
G660	CD3	PE-Cy5	UCHT1	555334	0037612	BD Biosciences	0.31	BDC backbone
G710	CD5	PE-Cy5.5	CD5-5D7	MHCD0518	2164661	Thermo Fisher	0.31	BDC backbone
R670	CD11c	APC	B-ly6	559877	0183298	BD Biosciences	5	BDC backbone
R730	CD27	APC-R700	M-T271	624348	8242909	BD Biosciences	0.63	BDC backbone
U500	CD40	BUV496	5C3	741159	everal pre-mixe	BD Biosciences	1.25	BDC backbone
U570	CD56	BUV563	NCAM16.2	565704	8065832	BD Biosciences	0.31	BDC backbone
U670	CD21	BUV661	B-ly4	741605	everal pre-mixe	BD Biosciences	0.31	BDC backbone
U740	CD163	BUV737	GHI/61	741863	everal pre-mixe	BD Biosciences	5	BDC backbone
U785	CD20	BUV805	2H7	612905	0290068	BD Biosciences	2.5	BDC backbone
V510	CD14	BV510	MPhiP9	624289	8241676	BD Biosciences	0.1	BDC backbone
V570	CD16	BV570	3G8	302036	B314477	Biolegend	1.25	BDC backbone
V605	CD38	BV605	HIT2	740401	everal pre-mixe	BD Biosciences	1.25	BDC backbone
V710	CD86	BV711	2331	563158	0246002	BD Biosciences	0.63	BDC backbone
V785	HLA-DR	BV786	G46-6	564041	0336621	BD Biosciences	0.15	BDC backbone
V450	CCR2	BV421	48607	564067	280237	BD Biosciences	5	Chemokine receptor 1 (CR1)
U395	CCR3	BUV395	5E8	743063	everal pre-mixe	BD Biosciences	2.5	Chemokine receptor 1 (CR1)
V655	CCR5	BV650	2D7/CCR5	740600	everal pre-mixe	BD Biosciences	2.5	Chemokine receptor 1 (CR1)
V750	CX3CR1	BV750	2A9-1	747376	everal pre-mixe	BD Biosciences	5	Chemokine receptor 1 (CR1)
G780	CCR1	PE-Cy7	5F10B29	362914	B303921	Biolegend	5	Chemokine receptor 1 (CR1)
R780	XCR1	APC-Fire750	S15046E	372608	B330906	Biolegend	5	Chemokine receptor 1 (CR1)
V450	CCR9	BV421	L053E8	358914	B317814	Biolegend	5	Chemokine receptor 2 (CR2)
U395	CCR8	BUV395	433H	747573	everal pre-mixe	BD Biosciences	1.25	Chemokine receptor 2 (CR2)
V655	CCR4	BV650	1G1	744140	everal pre-mixe	BD Biosciences	5	Chemokine receptor 2 (CR2)
V750	CXCR6	BV750	13B 1E5	747052	everal pre-mixe	BD Biosciences	5	Chemokine receptor 2 (CR2)
G780	CXCR3	PE-Cy7	G025H7	353720	B300691	Biolegend	1.25	Chemokine receptor 2 (CR2)
R780	CD19	APC-H7	SC25C1	560177	0126182	BD Biosciences	0.31	Chemokine receptor 2 (CR2) - BDC
R780	TIGIT	APC-Cy7	A15153G	372734	B329043	Biolegend	0.63	Chemokine receptor 2 (CR2) - TNK

Supplementary Table 3: Markers excluded for FlowSOM analysis per immune cell lineage.

T cell / NK cell backbone (TNK)				
**Panel**	TNK-CR1 and TNK-CR2	TNK-CR1 and TNK-CR2	TNK-CR1 and TNK-CR2	TNK-CR1 and TNK-CR2
**Lineage**	CD4 T cells	CD8 T cells	Innate T cells	NK cells
**Marker**	Viability	Viability	Viability	Viability
	CD1d:PBS57 tetramer	CD1d:PBS57 tetramer	CD3	CD1d:PBS57 tetramer
	CD3	CD3		CD3
	CD4	CD4		TCR Vd1
	CD8a	CD8a		TCR Vd2
	TCR Vd1	TCR Vd1		TCR Vg9
	TCR Vd2	TCR Vd2		TCR Va7.2
	TCR Vg9	TCR Vg9		HLA-DR

B cell / myeloid cell backbone (BDC)				
**Panel**	BDC-CR1	BDC-CR2	BDC-CR1	BDC-CR2
**Lineage**	Myeloid cells	Myeloid cells	B cells	Bcells
**Marker**	Viability	Viability	Viability	Viability
	CD3	CD3	CADM1	CADM1
	CD20	CD19	CD141	CD141
	CD21	CD20	CD123	CD123
	IgD	CD21	FcER1a	FcER1a
		IgD	CD88	CD88
			CD3	CD3
			CD56	CD19
			CD163	CD56
			CD14	CD163
			CD16	CD14
			HLA-DR	CD16
				HLA-DR

Supplementary Table 4: FlowSOM clusters with potential immune cell contaminants derived from other lineages removed from subsequent analysis.

Contaminatiing immune populations - FlowSOM clusters removed from subsequent analysis												
**Panel**	BDC-CR1	BDC-CR2	BDC-CR1	BDC-CR2	TNK-CR1	TNK-CR2	TNK-CR1	TNK-CR2	TNK-CR1	TNK-CR2	TNK-CR1	TNK-CR2
**Lineage**	B cells	B cells	Myeloid cells	Myeloid cells	CD4 T cells	CD4 T cells	CD8 T cells	CD8 T cells	Innate T cells	Innate T cells	NK cells	NK cells
**Clusters**					23	11	18	6	20	17	1	1
	all clusters included	all clusters included	all clusters included	all clusters included	38	22	28	34			5	2
											7	3
											9	4
											11	7
											18	8
											20	14
											22	15
											23	16
											25	17
											29	19

Supplementary Table 5: Buffers and cell culture media used in this study.

Item	Vendor	Catalog#
RPMI w/o phenol red	Thermo Fisher Scientific	A14576DK
Dimethyl sulfoxide (DMSO)	Sigma-Aldrich	D5879-100ml
Newborn Calf Serum, Heat Inactivated (HINCS)	R&D Systems	S11250H
Brilliant Stain Buffer Plus	BD Biosciences	566385
True-Stain Monocyte Blocker™	Biolegend	426103
RPMI 1640 medium, no glutamine	Thermo Fisher Scientific	21870092
Fetal Bovine Serum, heat Inactivated (FBS)	Thermo Fisher Scientific	10438026
Penicillin-Streptomycin-Glutamine (100X)	Thermo Fisher Scientific	10378016
Benzonase	Millipore Sigma	71205-25KUN
DPBS, no calcium, no magnesium	Thermo Fisher Scientific	14190-250
Human BD Fc Block™ (250ug)	BD Biosciences	564220
20% Formaldehyde	Tousimis	1008B
