# Supplementary material for: Immune phenotypes that predict COVID-19 severity
Source: Res Sq. 2022 Mar 10:rs.3.rs-1378671. Preprint. [Version 1] doi: 10.21203/rs.3.rs-1378671/v1 (PMC8923110; doi:10.21203/rs.3.rs-1378671/v1)
Supplement: 1 [file NIHPPRS1378671V1-supplement-1.pdf]

Supplementary Data

Title: Immune phenotypes that predict COVID-19 severity

Thomas Liechti[1], Yaser Iftikhar[1], Massimo Mangino[2,3], Margaret Beddall[1], Charles W. Goss[4], Jane A. O'Halloran[5], Philip Mudd[6], Mario Roederer[1]

[1]ImmunoTechnology Section, Vaccine Research Center, NIAID, NIH, USA
[2]Department of Twin Research & Genetic Epidemiology, King's College of London, London, UK
[3] NIHR Biomedical Research Centre at Guy's and St Thomas' Foundation Trust, London SE1 9RT, UK
[4]Division of Biostatistics, Washington University School of Medicine, St. Louis, MO, USA,
[5]Division of Infectious Diseases, Department of Internal Medicine, Washington University School of Medicine, St. Louis, MO, USA
[6]Department of Emergency Medicine, Washington University School of Medicine, St. Louis, MO, USA

Keywords
SARS-CoV2, COVID-19, Immunophenotyping, Chemokine Receptors, High-dimensional flow cytometry

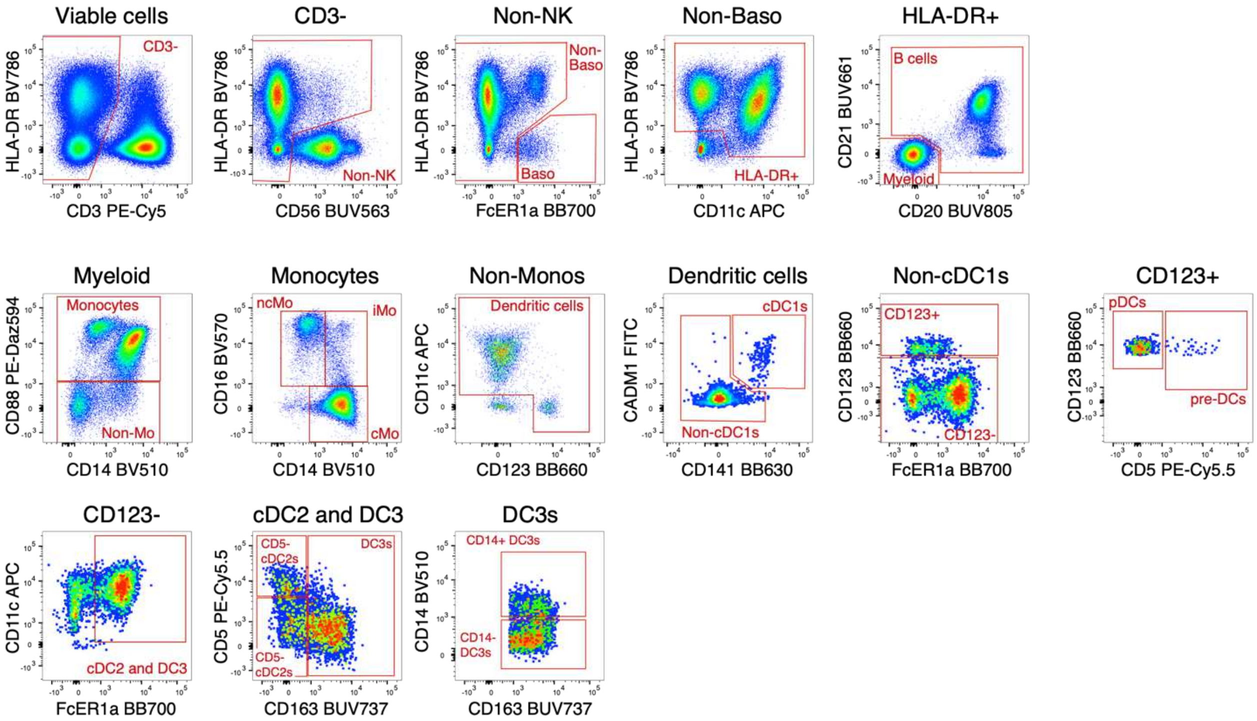

Supplementary Data 1: Gating of myeloid cells (Monocytes/Dendritic cells)

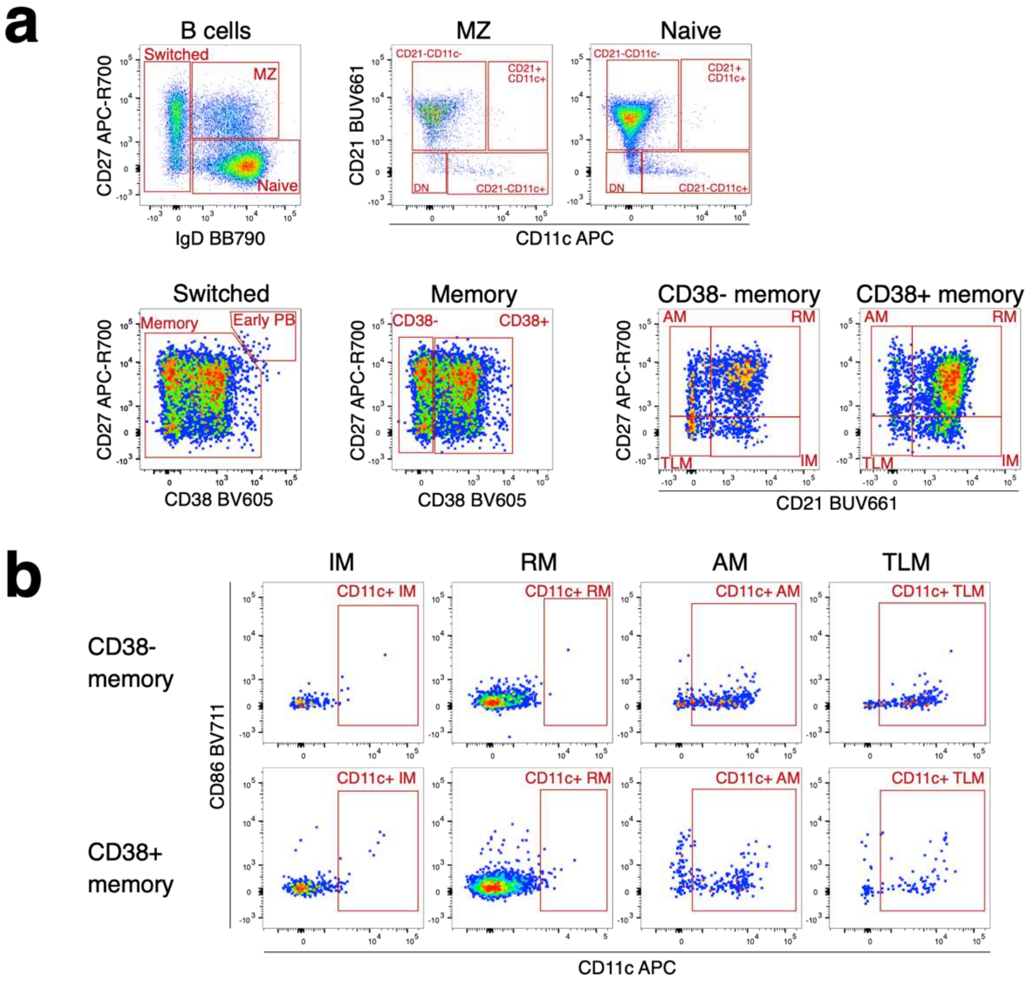

Supplementary Data 2: Gating of B cell subsets

Gating of **a)** B cell subsets and **b)** CD11c$^+$ B cells within memory B cell subsets (Intermediate memory, IM; Resting memory, RM; Activated memory, AM; Tissue-like memory, TLM) of CD38$^-$ (top row) and CD38$^+$ (bottom row) memory B cells is depicted.

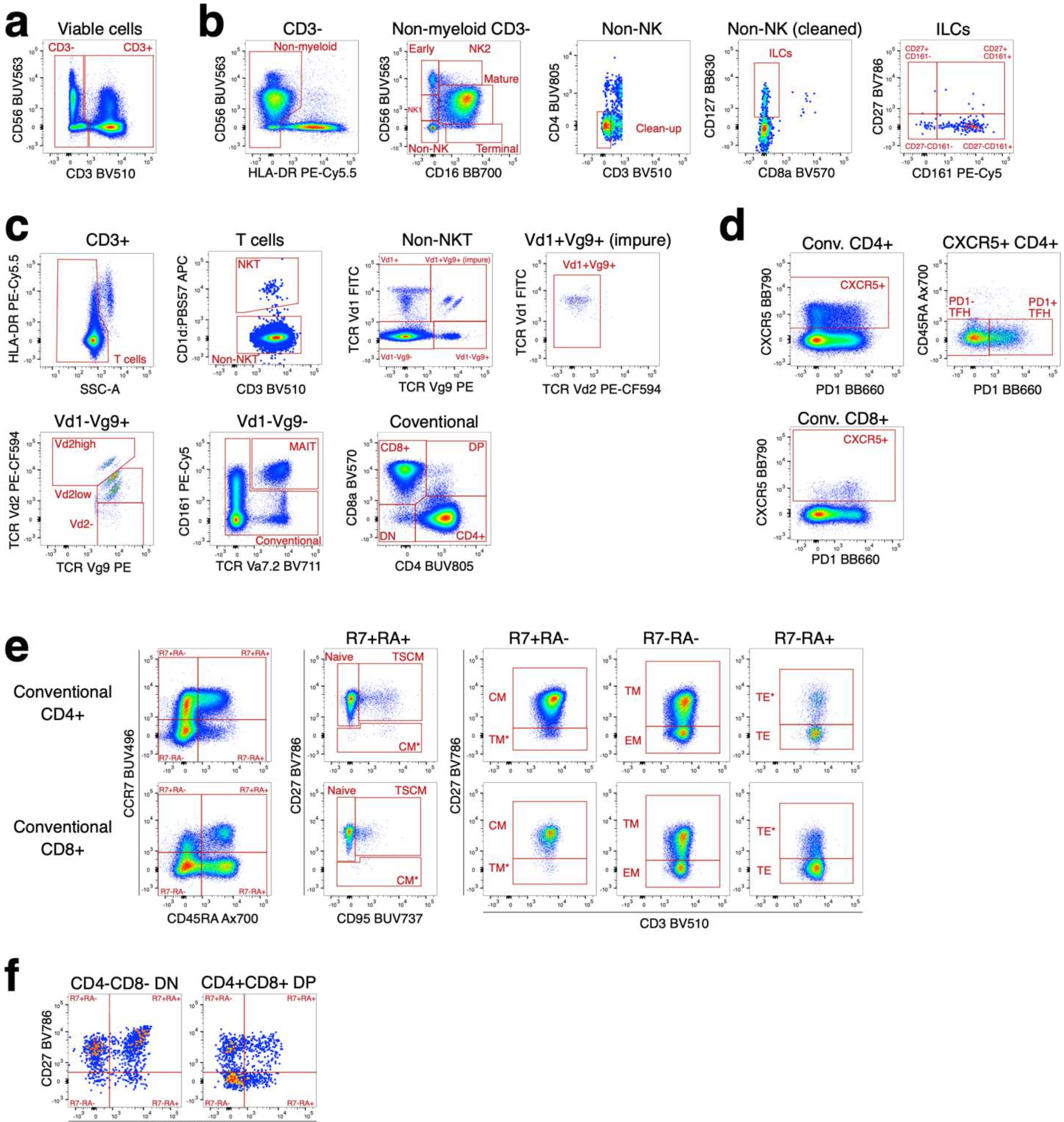

Supplementary Data 3: Gating of innate-like and conventional T cell subsets and NK cells

a) Definition of CD3- and CD3+ cells is shown. **b)** Gating of NK cells and innate lymphoid cells (ILCs) is depicted. HLA-DR expressing cells were excluded prior to

defining NK cells based on CD56 and CD16. We further removed residual contaminating cells within CD56$^-$CD16$^-$ cells based on CD4 and CD8 prior to defining CD127 expressing ILCs and subsets of ILCs based on CD27 and CD161 expression. **c)** Definition of unconventional and conventional T cells is shown after excluding residual myeloid cells based on SSC-A and expression of HLA-DR. **d)** Definition of CD4$^+$ and CD8$^+$ T cell memory subsets is depicted. **f)** We further defined subsets from CD4$^-$CD8$^-$ double-negative (DN) and CD4$^+$CD8$^+$ double-positive (DP) T cells based on expression of CD27 and CD45RA.

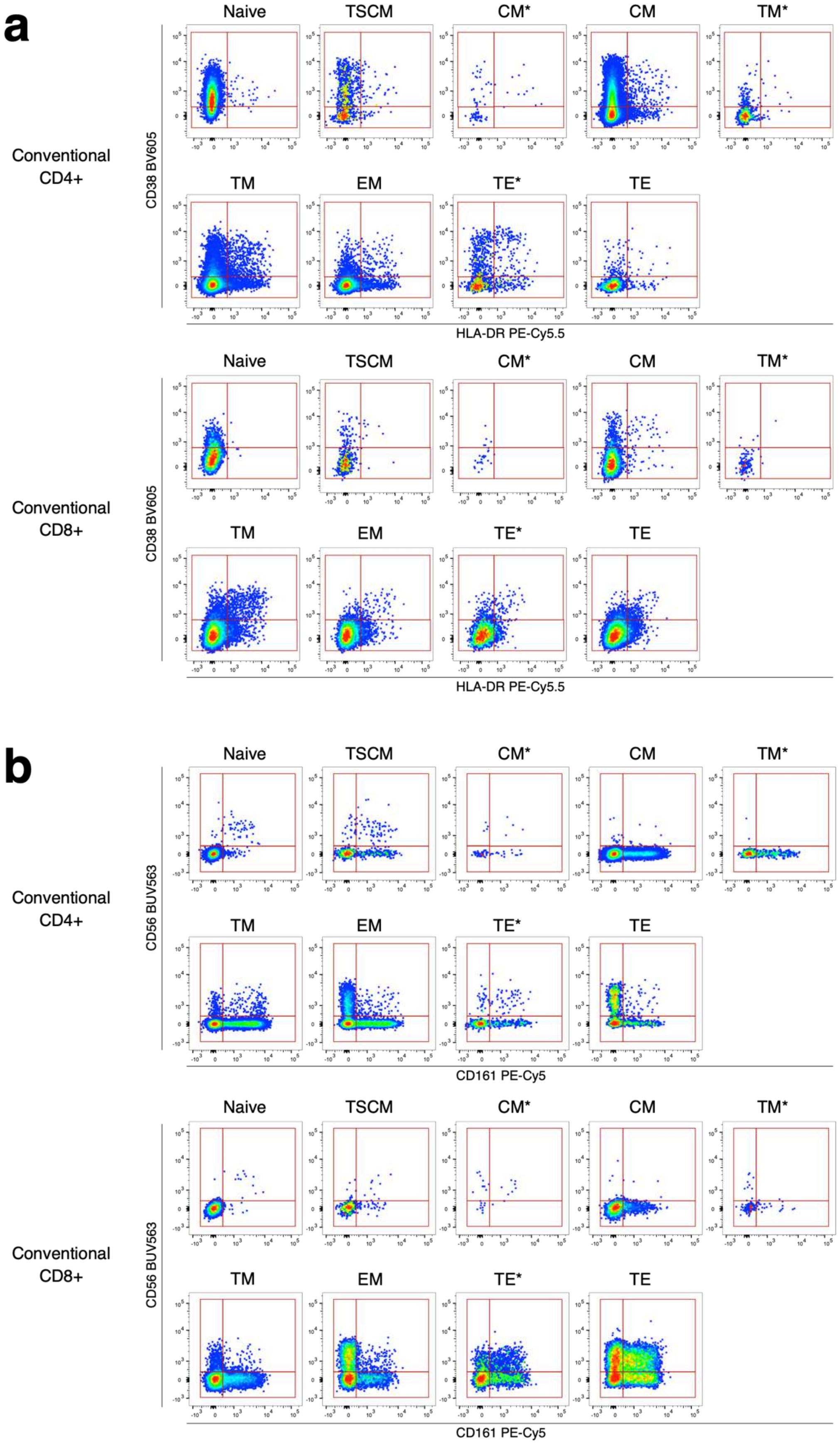
a
Naive
TSCM
CM*
CM
TM*
Conventional CD4+
CD38 BV605
TM
EM
TE*
TE
HLA-DR PE-Cy5.5
Naive
TSCM
CM*
CM
TM*
Conventional CD8+
CD38 BV605
TM
EM
TE*
TE
HLA-DR PE-Cy5.5
b
Naive
TSCM
CM*
CM
TM*
Conventional CD4+
CD56 BUV563
TM
EM
TE*
TE
CD161 PE-Cy5
Naive
TSCM
CM*
CM
TM*
Conventional CD8+
CD56 BUV563
TM
EM
TE*
TE
CD161 PE-Cy5

Supplementary Data 4: Gating conventional T cell subsets based on CD38, HLA-DR, CD56 and CD161

Definition of subsets within conventional $CD4^+$ and $CD8^+$ naïve and memory T cell populations based on **a)** CD38 and HLA-DR or **b)** CD56 and CD161 expression.

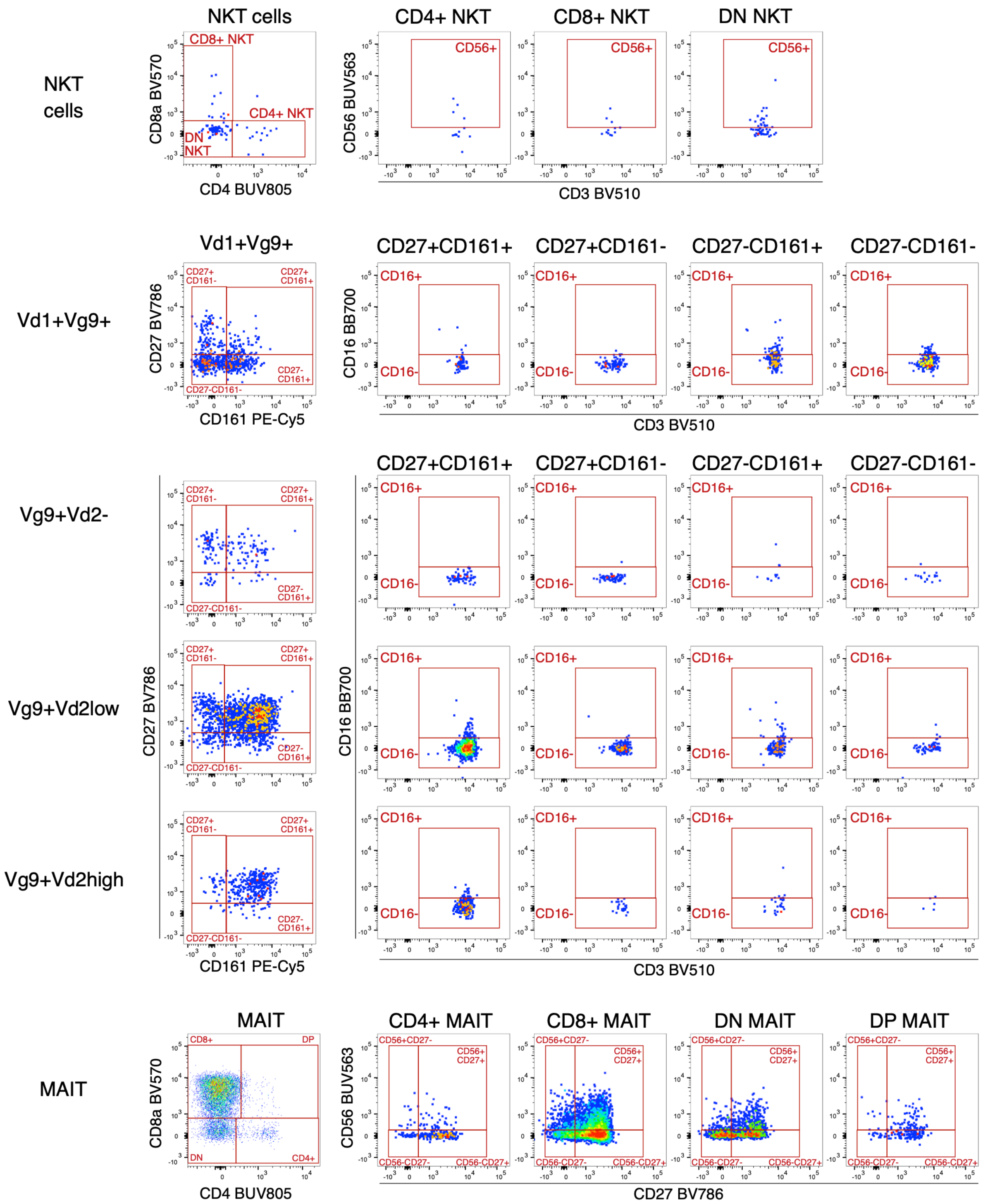

Supplementary Data 5: Gating of several differentiation stages within innate-like T cell subsets

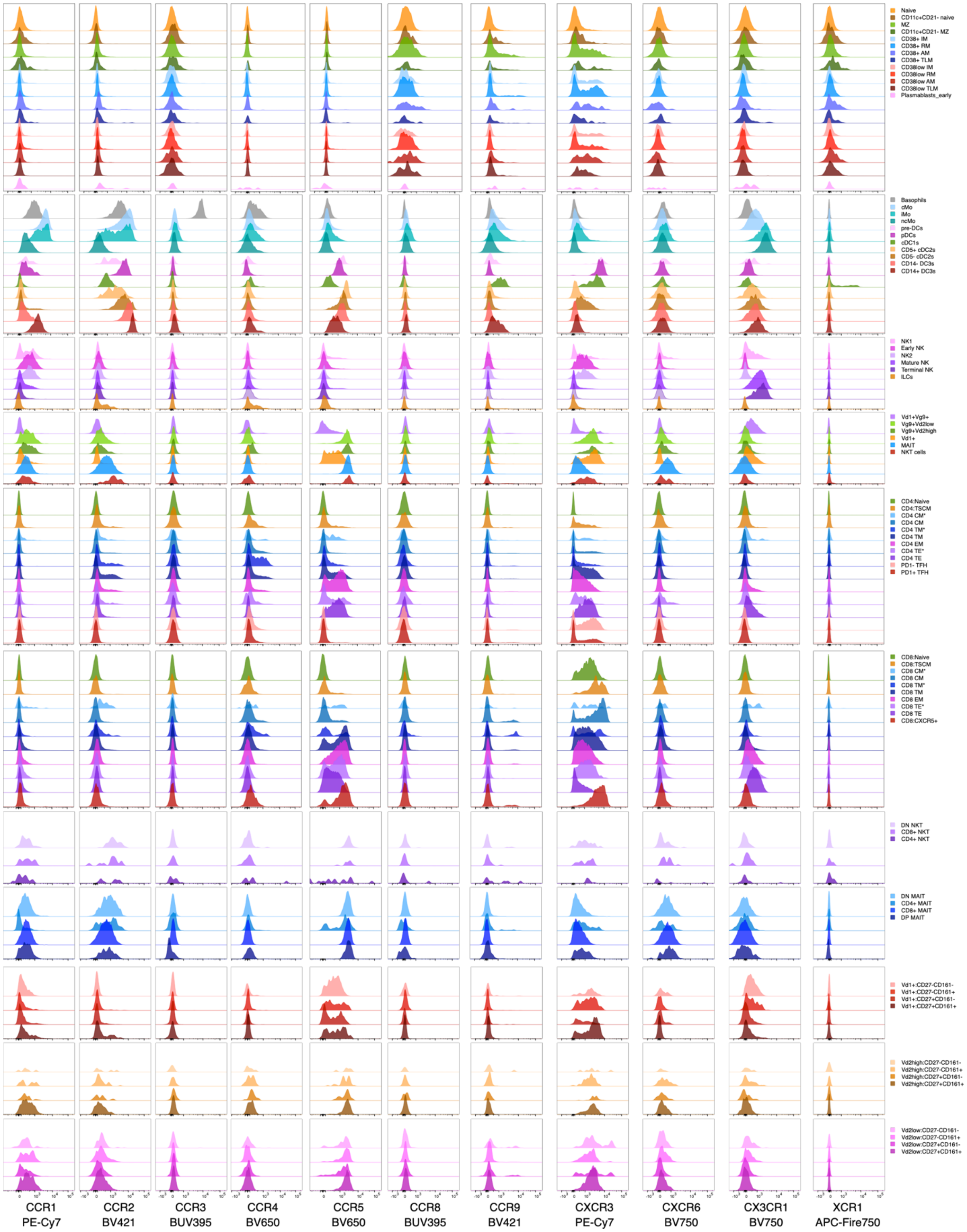
Naive
CD11c+CD21- naive
MZ
CD11c+CD21- MZ
CD38+ IM
CD38+ RM
CD38+ AM
CD38+ TLM
CD38low IM
CD38low RM
CD38low AM
CD38low TLM
Plasmablasts_early
Basophils
cMo
iMo
ncMo
pre-DCs
pDCs
cDC1s
CD5+ cDC2s
CD5- cDC2s
CD14- DC3s
CD14+ DC3s
NK1
Early NK
NK2
Mature NK
Terminal NK
ILCs
Vd1+Vg9+
Vg9+Vd2low
Vg9+Vd2high
Vd1+
MAIT
NKT cells
CD4:Naive
CD4:TSCM
CD4 CM*
CD4 CM
CD4 TM*
CD4 TM
CD4 EM
CD4 TE*
CD4 TE
PD1- TFH
PD1+ TFH
CD8:Naive
CD8:TSCM
CD8 CM*
CD8 CM
CD8 TM*
CD8 TM
CD8 EM
CD8 TE*
CD8 TE
CD8:CXCR5+
DN NKT
CD8+ NKT
CD4+ NKT
DN MAIT
CD4+ MAIT
CD8+ MAIT
DP MAIT
Vd1+:CD27-CD161-
Vd1+:CD27-CD161+
Vd1+:CD27+CD161-
Vd1+:CD27+CD161+
Vd2high:CD27-CD161-
Vd2high:CD27-CD161+
Vd2high:CD27+CD161-
Vd2high:CD27+CD161+
Vd2low:CD27-CD161-
Vd2low:CD27-CD161+
Vd2low:CD27+CD161-
Vd2low:CD27+CD161+
CCR1 PE-Cy7
CCR2 BV421
CCR3 BUV395
CCR4 BV650
CCR5 BV650
CCR8 BUV395
CCR9 BV421
CXCR3 PE-Cy7
CXCR6 BV750
CX3CR1 BV750
XCR1 APC-Fire750

Supplementary Data 6: Expression of chemokine receptors

Shown is the expression of chemokine receptors on all main lineages/immune subsets as overlaid histograms. Data derives from one healthy donor.

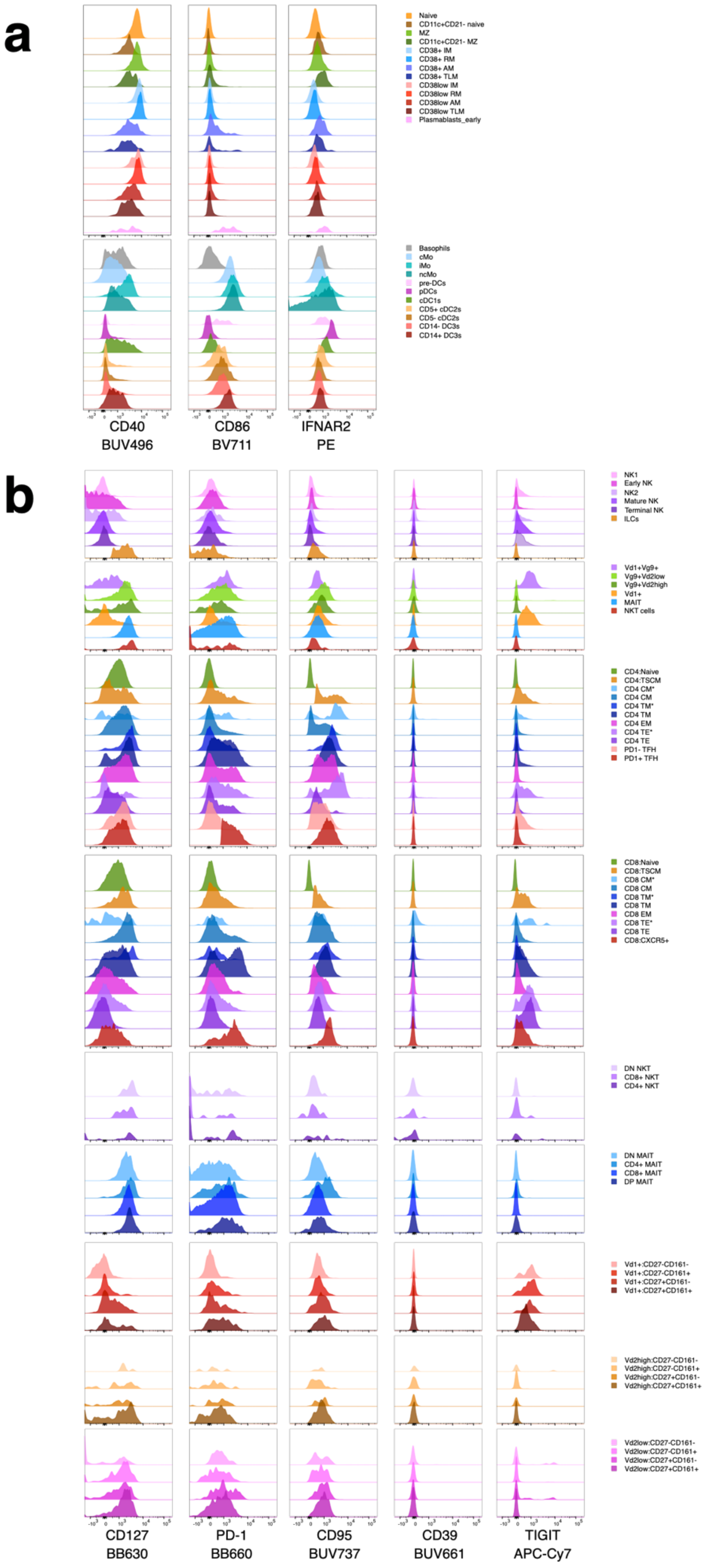

a
Naive
CD11c+CD21- naive
MZ
CD11c+CD21- MZ
CD38+ IM
CD38+ RM
CD38+ AM
CD38+ TLM
CD38low IM
CD38low RM
CD38low AM
CD38low TLM
Plasmablasts_early
Basophils
cMo
iMo
ncMo
pre-DCs
pDCs
cDC1s
CD5+ cDC2s
CD5- cDC2s
CD14- DC3s
CD14+ DC3s
CD40
BUV496
CD86
BV711
IFNAR2
PE
b
NK1
Early NK
NK2
Mature NK
Terminal NK
ILCs
Vd1+Vg9+
Vg9+Vd2low
Vg9+Vd2high
Vd1+
MAIT
NKT cells
CD4:Naive
CD4:TSCM
CD4 CM*
CD4 CM
CD4 TM*
CD4 TM
CD4 EM
CD4 TE*
CD4 TE
PD1- TFH
PD1+ TFH
CD8:Naive
CD8:TSCM
CD8 CM*
CD8 CM
CD8 TM*
CD8 TM
CD8 EM
CD8 TE*
CD8 TE
CD8:CXCR5+
DN NKT
CD8+ NKT
CD4+ NKT
DN MAIT
CD4+ MAIT
CD8+ MAIT
DP MAIT
Vd1+:CD27-CD161-
Vd1+:CD27-CD161+
Vd1+:CD27+CD161-
Vd1+:CD27+CD161+
Vd2high:CD27-CD161-
Vd2high:CD27-CD161+
Vd2high:CD27+CD161-
Vd2high:CD27+CD161+
Vd2low:CD27-CD161-
Vd2low:CD27-CD161+
Vd2low:CD27+CD161-
Vd2low:CD27+CD161+
CD127
BB630
PD-1
BB660
CD95
BUV737
CD39
BUV661
TIGIT
APC-Cy7

Supplementary Data 7: Expression of functional receptors

Shown is the expression of functional receptors on main lineages/immune subsets as overlaid histograms. Markers are panel specific. Markers only measured with the **a)** B cell/myeloid cell or **b)** T cell/NK cell panel backbone are shown as highlighted in supplementary table 2. Data derives from one healthy donor.

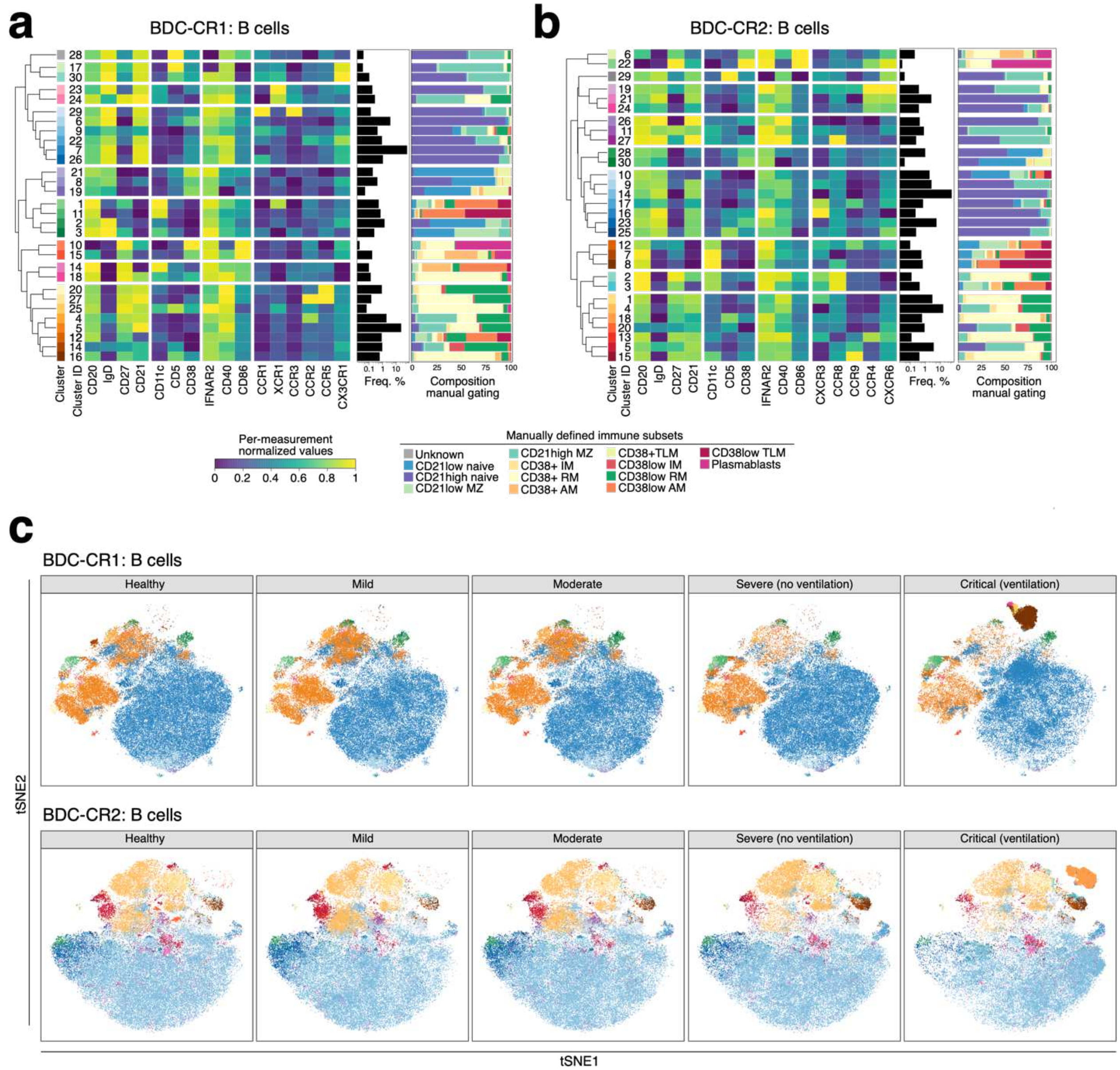

Supplementary Data 8: FlowSOM analysis for B cells

Heatmaps show per-measurement normalized median fluorescence intensity based on trimmed 1-99% percentile values for each FlowSOM cluster (rows). Only markers included in clustering (columns) are sown. Bar on left shows coloring of each FlowSOM cluster and FlowSOM clusters were clustered based on similarity of MFI values using hierarchical clustering (indicated by dendrogram and gap between rows). Bar graph in

the middle shows the frequency of each cluster and bar graph on the right the composition of each cluster based on manual gating annotation. Heatmaps for panels **a)** CR1 and **b)** CR2 are shown. **c)** tSNE plots for CR1 (top) and CR2 (bottom) panel are shown delineated based on COVID-19 severity group. Dots are colored based on FlowSOM cluster annotation. Each tSNE plot contains 50’000 randomly subsampled cells and not equally distributed across each individual sample.

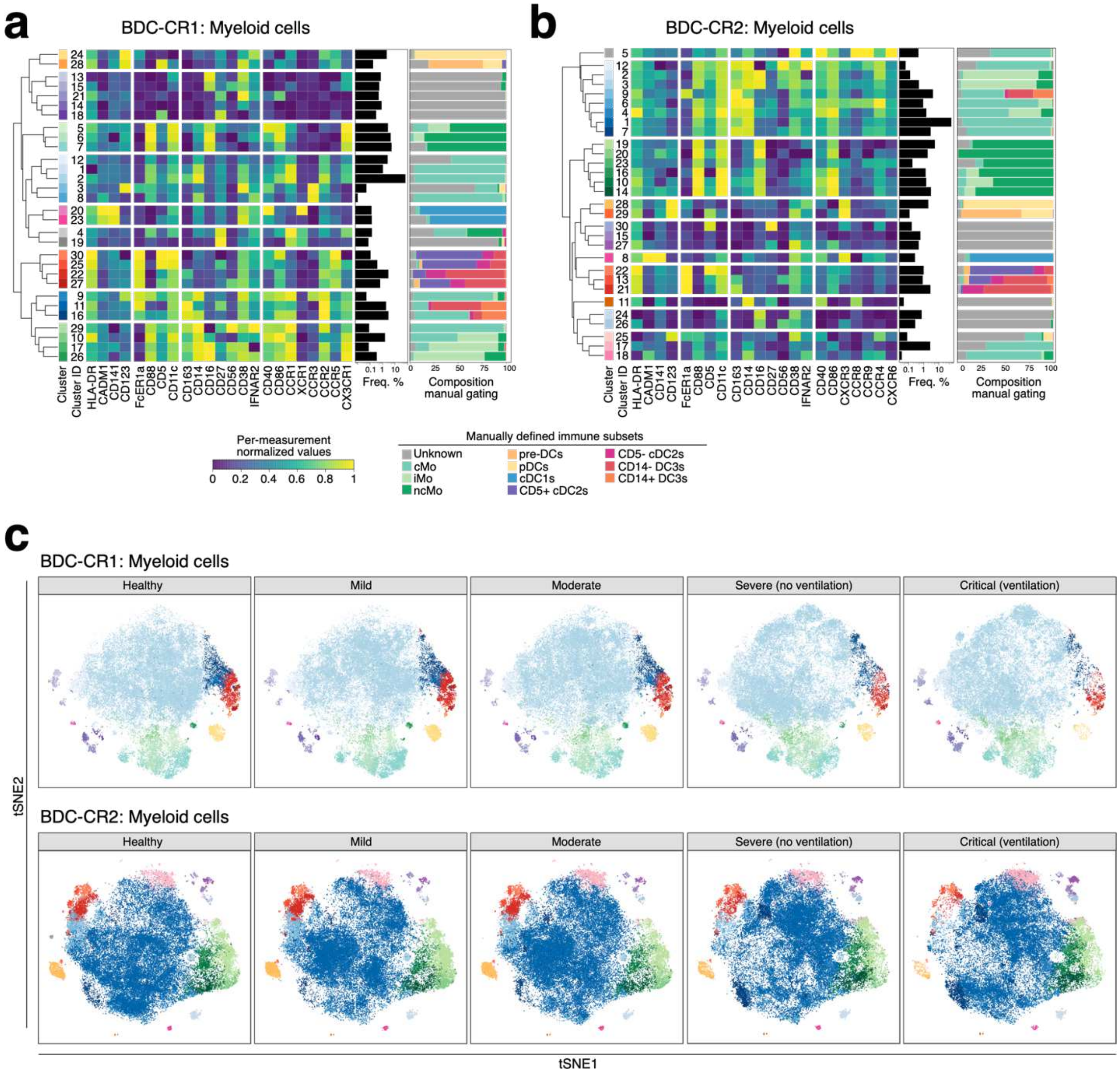

Supplementary Data 9: FlowSOM analysis for myeloid cells

Same as Supplementary Data 8.

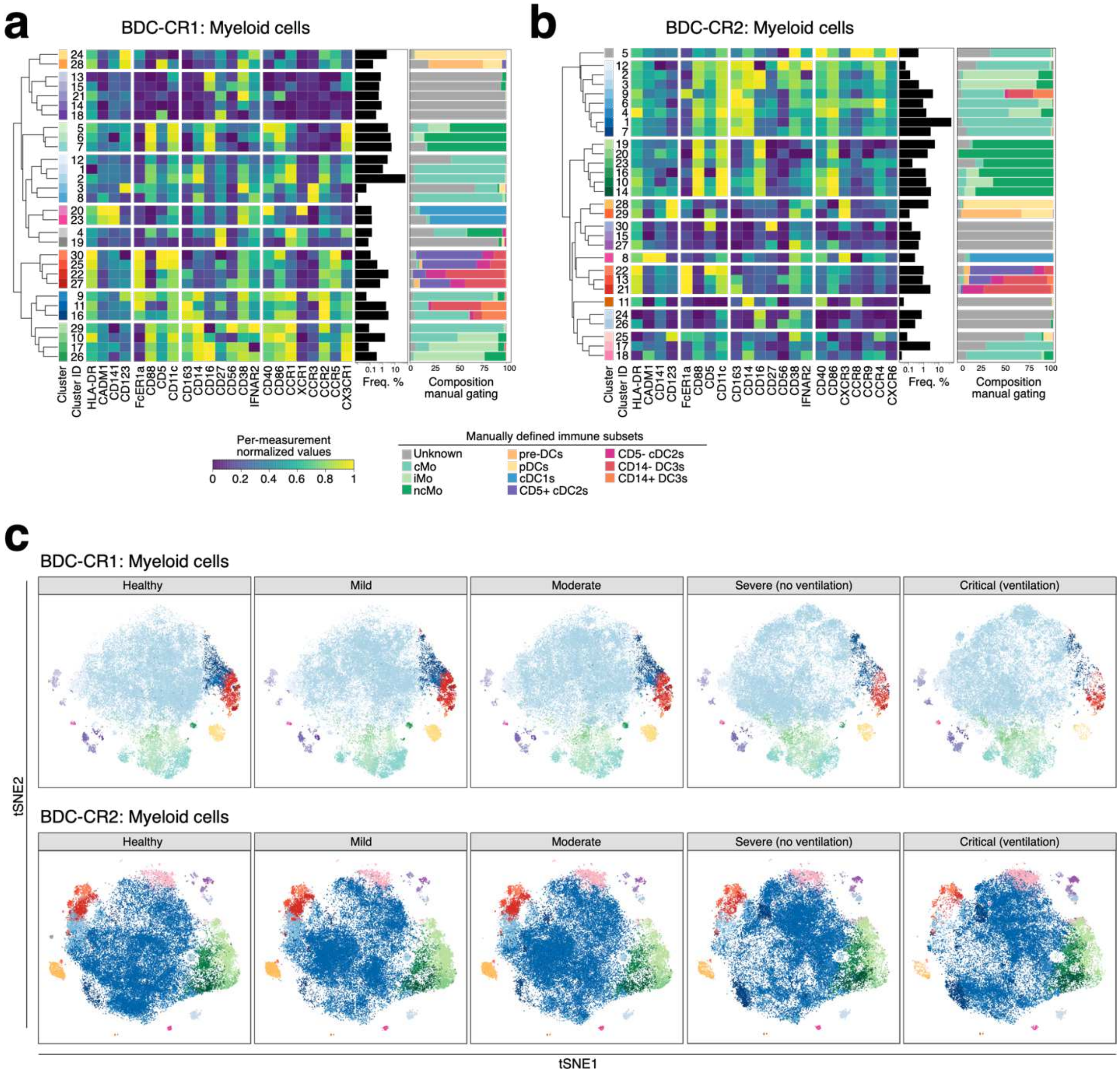

Supplementary Data 10: FlowSOM analysis for CD4 T cells

Same as Supplementary Data 8.

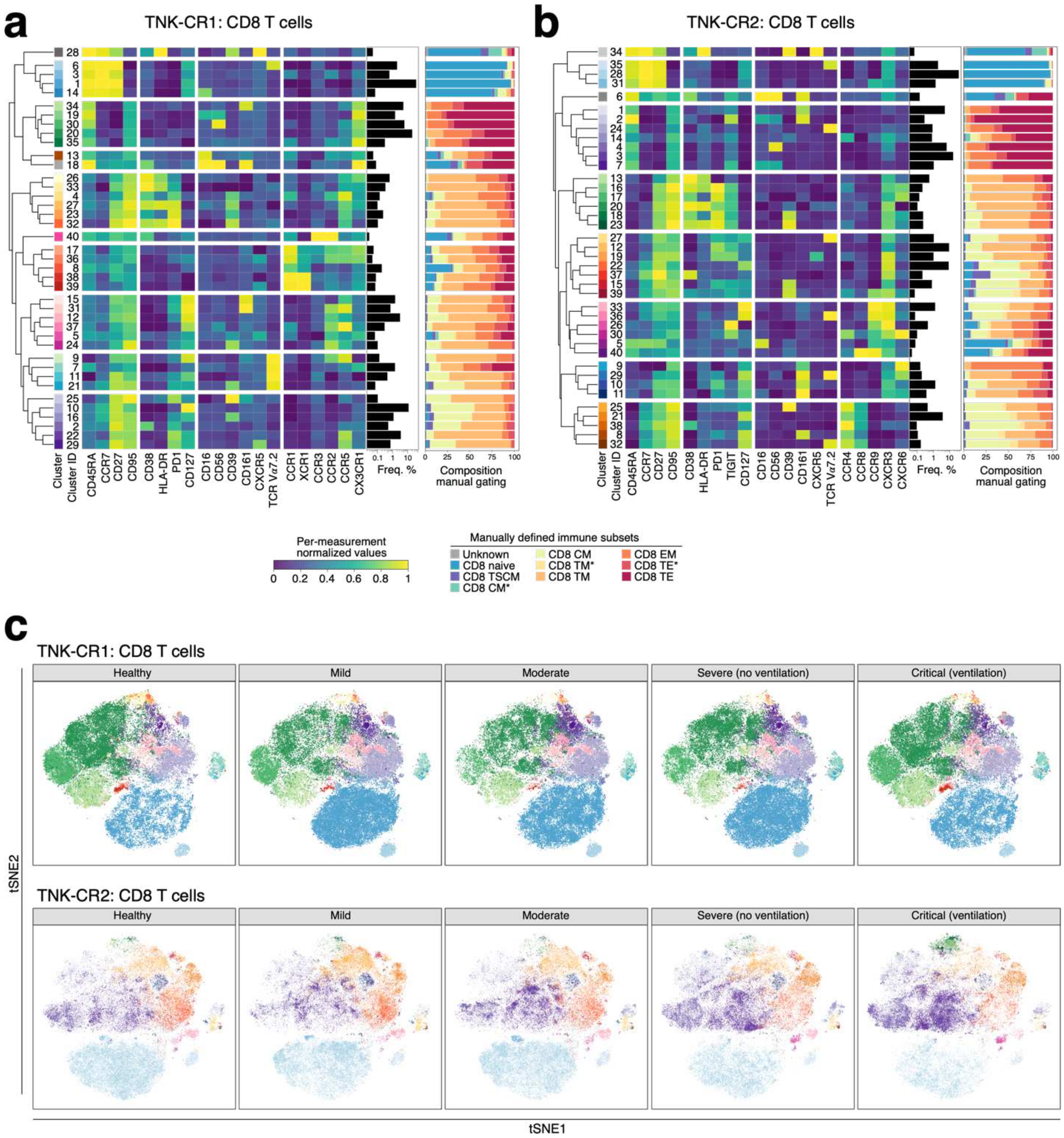

Supplementary Data 11: FlowSOM analysis for CD8 T cells

Same as Supplementary Data 8.

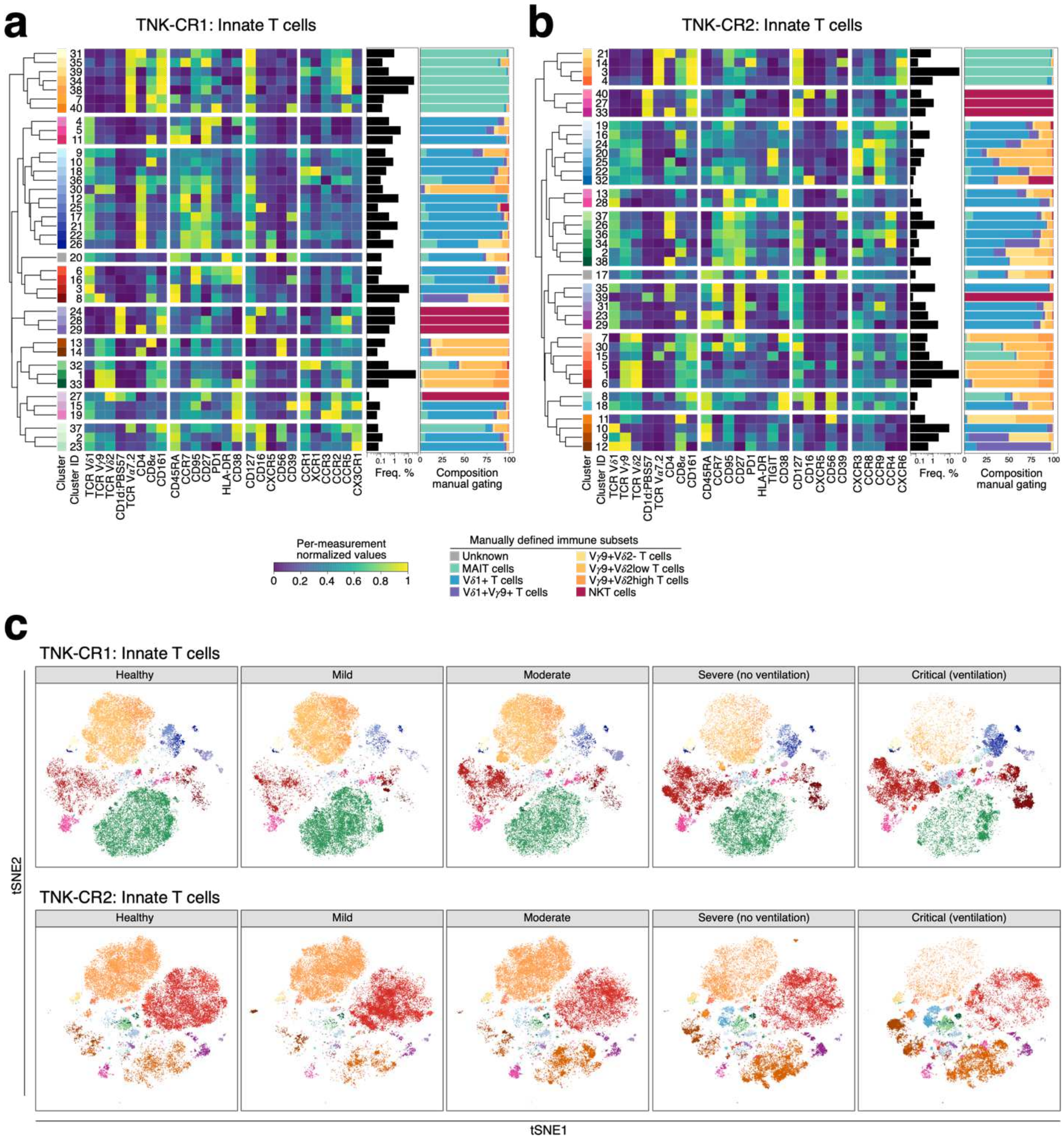

Supplementary Data 12: FlowSOM analysis for innate-like T cells

Same as Supplementary Data 8. From each group 27583 cells were included for tSNE computation.

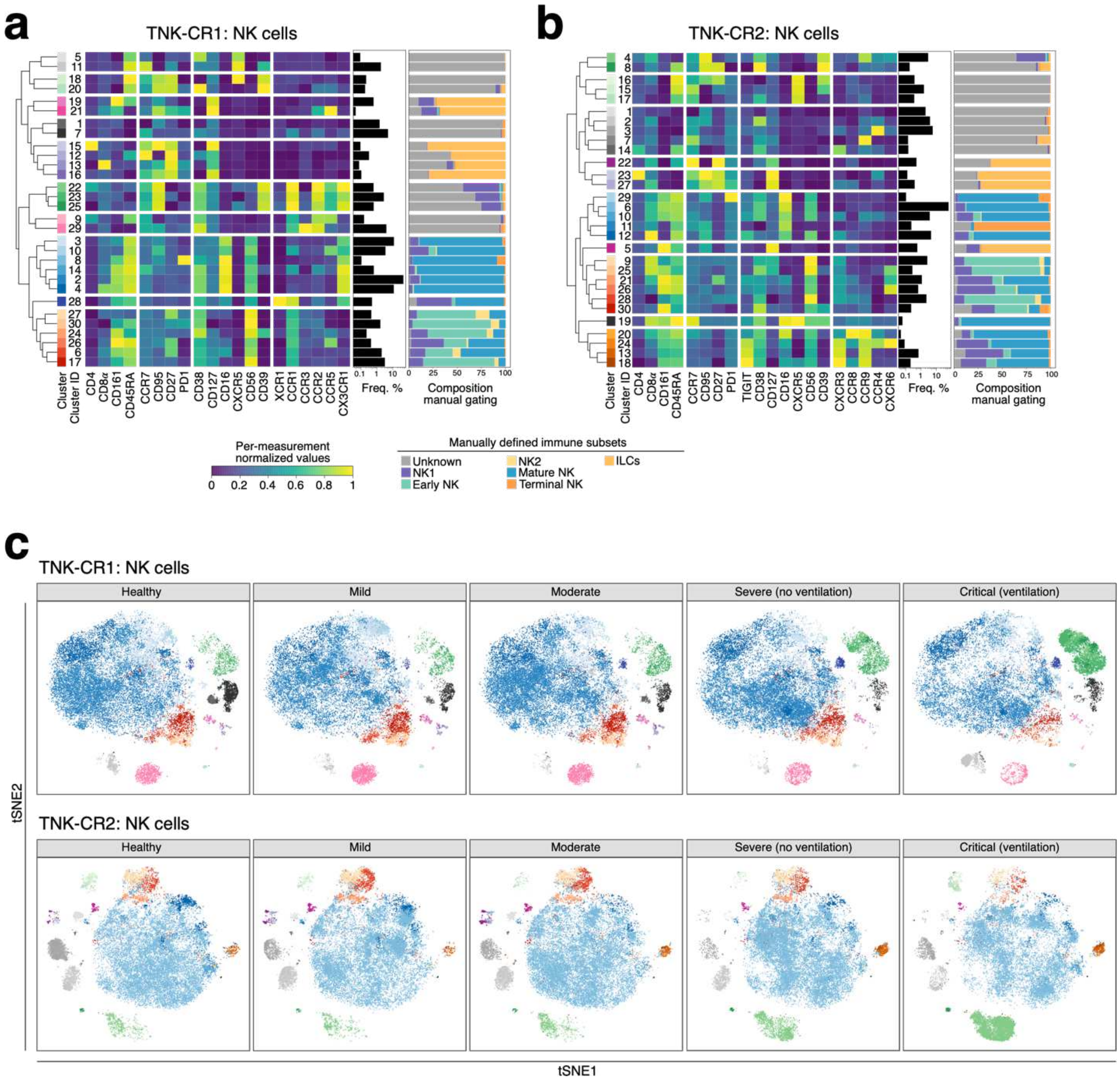

Supplementary Data 13: FlowSOM analysis for NK cells

Same as Supplementary Data 8. From each group 25000 cells were included for tSNE computation.

Supplementary Files

This is a list of supplementary files associated with this preprint. Click to download.

- 220218Supplementarytables.pdf
